# Effects of Obligate Heterofermentative Lactic Acid Bacteria Alone or in Combination on the Conservation of Sugarcane Silage

**DOI:** 10.3389/fmicb.2021.643879

**Published:** 2021-05-10

**Authors:** Ana Luiza Mendonça Gomes, Antônio Vinicius Iank Bueno, Milene Puntel Osmari, Juliana Machado, Luiz Gustavo Nussio, Clóves Cabreira Jobim, João Luiz Pratti Daniel

**Affiliations:** ^1^Department of Animal Science, State University of Maringá, Maringá, Brazil; ^2^Department of Animal Science and Rural Development, Federal University of Santa Catarina, Florianópolis, Brazil; ^3^Department of Animal Science, Luiz de Queiroz College of Agriculture, University of São Paulo, Piracicaba, Brazil

**Keywords:** aerobic stability, acetic acid, gas loss, *Lentilactobacillus buchneri*, *Lentilactobacillus hilgardii*

## Abstract

Our objective was to determine the effects of two strains of obligate heterofermentative bacteria, alone or in combination, on the fermentation profile, gas production kinetics, chemical composition, and aerobic stability of sugarcane silage. A plot of sugarcane was manually harvested, mechanically chopped and treated with: distilled water (5 mL kg^–1^; Control), *Lentilactobacillus hilgardii* CNCM I-4785 [3 × 10^5^ colony-forming units (cfu) g^–1^; LH], *Lentilactobacillus buchneri* NCIMB 40788 (3 × 10^5^ cfu g^–1^; LB), and LH+LB (1.5 × 10^5^ cfu g^–1^ of each strain). Treated forages were packed into 1.96-L gas-tight silos (0.40 porosity) and stored at 25 ± 1.5°C for 70 days (4 replicates per treatment). All heterolactic inoculants were effective to increase acetic acid concentration and inhibit yeast metabolism, as treated silages had lower formation of ethanol, ethyl esters and gas during fermentation. Lower fungal development spared soluble carbohydrates, consequently resulting in silages with higher *in vitro* digestibility. Nevertheless, *L. buchneri* was the most effective strain to extend the aerobic stability of sugarcane silage (based on both temperature and pH rise). The use of *L. buchneri* alone or in combination with *L. hilgardii*, applied at 3 × 10^5^ cfu g^–1^, is a feasible strategy to inhibit yeast metabolism and increase the nutritional quality of sugarcane silage.

## Introduction

In tropical areas, sugarcane (*Saccharum officinarum* L.) crop is characterized by a high dry matter (DM) yield (>30 t DM/ha) within one harvest and a suitable nutritive value at maturity (48 h DM digestibility > 600 g kg^–1^; Daniel et al., 2013b), enabling high animal stocking rates. Although the maximum nutritive value of sugarcane matches pasture shortage during dry season, sugarcane has been ensiled to avoid daily harvesting, prevent crop lodging and prolong field lifespan by better agronomic management (Daniel et al., 2019).

Despite its high fermentation capability (appropriate DM content, low buffering capacity and high content of soluble sugar at ensiling; fermentability coefficient ∼170), sugarcane conserved by natural fermentation has high fermentation loss (up to 300 g kg^–1^ of ensiled DM), due to the formation of ethanol and CO_2_ by yeast metabolism (Kung and Stanley, 1982; Pedroso et al., 2005; Ávila et al., 2010). Additionally, high yeast counts in sugarcane silages contribute to aerobic deterioration after feedout, increasing total nutrient losses (Ávila et al., 2012).

A feasible strategy to reduce yeast activity in sugarcane silage, both during storage and feedout phases, is the application of chemical or microbial additives (Pedroso et al., 2011). In general, both chemical additives with antifungal power and obligate heterofermentative lactic acid bacteria (LAB) are effective to protect sugarcane silage against yeast detrimental effects (Daniel et al., 2015b); whereas bacterial inoculants are cheaper than chemical additives (Kung et al., 2003).

Different obligate heterofermentative LAB have been launched in the market during the last two decades (Muck, 2013; Ávila et al., 2014; Daniel et al., 2015a). More recently, the combination of obligate heterofermentative LAB applied at ≥ 3 × 10^5^ cfu g^–1^ has shown potential benefits in studies with whole-plant corn silage and high moisture corn (Drouin et al., 2019; da Silva et al., 2021; Ferrero et al., 2021). Besides, Ferrero et al. (2021) reported that combination of *L. hilgardii* and *L. buchneri* improved the aerobic stability earlier than that inoculants alone, due to a reduction in yeast population. Whist the effects of single strains of heterolactic bacteria have been reported in different trials, the associative effect of obligate heterofermentative LAB in sugarcane silage is unknown. Additionally, most studies on the effects of obligate heterofermentative LAB in sugarcane silage evaluated relatively low application rates (≤1 × 10^5^ cfu g^–1^) compared to doses marketed for different crops in the United States and Europe, which might contribute to controversial benefits of obligate heterofermentative LAB on the conservation of sugarcane silage (Schmidt, 2009; Rabelo et al., 2019).

Thus, the objective of this study was to compare the effectiveness of two strains of obligate heterofermentative LAB [one strain of *Lentilactobacillus buchneri* NCIMB 40788 (isolated from corn silage) and one strain of *Lentilactobacillus hilgardii* CNCM I-4785 (isolated from sugarcane silage)], alone or in combination, on the fermentation traits, microbial counts, fermentation losses, gas production kinetics and nutritional value of sugarcane silage. We hypothesize that either *L. buchneri* and *L. hilgardii* alone or in combination, inoculated at 3 × 10^5^ cfu g^–1^, would be effective to mitigate yeast detrimental effects in sugarcane silage, but expected that combination of *L. hilgardii* and *L. buchneri* would abate the gas production sooner.

## Materials and Methods

### Ensiling

Sugarcane variety CTC-25 was manually harvested from one plot at the State University of Maringá (Maringá, Brazil) after 10 months of regrowth (2nd cut) and chopped in a stationary cutter to a theoretical cut length of 8 mm. At harvest, sugarcane DM was 307 g kg^–1^ fresh matter (FM) and the content of soluble solids in the stalk juice was 24° Brix, indicating that the crop was mature. Chopped sugarcane was divided in four piles (8 kg fresh matter per pile). Piles were treated with: Control (without additive; 5 mL kg^–1^ of distilled water), *Lentilactobacillus hilgardii* CNCM I-4785 [3 × 10^5^ colony-forming units (cfu) g^–1^; LH], *Lentilactobacillus buchneri* NCIMB 40788/CNCM I-4323 (3 × 10^5^ cfu g^–1^, LB), and LH+LB (1.5 × 10^5^ cfu g^–1^ of *L. hilgardii* + 1.5 × 10^5^ cfu g^–1^ of *L. buchneri*). *L. hilgardii* and *L. buchneri* strains lyophilized and packed in aluminized pouches were donated by Lallemand SAS (Blagnac, France).

Afterward, treated forages were packed (0.40 porosity; Pitt, 1986) into 1.96-L gas-tight silos (∼1,280 g per silo) (Daniel and Nussio, 2015) and stored for 70 days (4 replicates per treatment). Once jar caps sealed, silos were stored in a room with controlled temperature (25 ± 1.5°C).

### Gas Production Kinetics and Fermentation Losses

The internal pressure of the silos was measured by using a pressure transducer coupled to a data logger (Daniel and Nussio, 2015). Pressure measures were performed twice daily during the first 3 days of storage, once daily until the end of the third week, every 3 days until the end of 2 months, and then once a week until 70 days. Pressure values were converted to volume (Daniel and Nussio, 2015) and the cumulative gas production per kg of DM was fitted with an exponential 1-pool model [*Gt* = (*G*×(1−*exp*^−(*k*×*t*)^))] to estimate the fractional rate of gas production (k) and gas pool (G) (Daniel et al., 2016). Gas emission was obtained by converting gas volume to CO_2_ mass, considering that 1 L of silage gas has 0.99 L of CO_2_ and that 1 L of CO_2_ has 1.96 g of CO_2_ (Daniel and Nussio, 2015). Gas loss also was determined by gravimetry, as the difference of silo mass at ensiling and at opening. Dry matter loss during fermentation was determined by the difference between forage DM mass at ensiling and silage DM mass at opening. Silage DM content used in this calculation was corrected for loss of volatile compounds, as described below.

### Laboratory Analysis

At silo opening, silage sub-samples were homogenized with distilled water to prepare aqueous extract (1:10; Kung et al., 1984). Aqueous extracts were used for measuring pH, microbial counts enumerated on selective agar media, and fermentation end-products.

Microbial counts [lactic acid bacteria (LAB), yeasts and molds] were evaluated in a serial dilution of the aqueous extract. Microorganisms were enumerated in Petri dishes with selective media. Malt Extract Agar (M137, Himedia, 632 Mumbai, India) acidified to pH 3.5 with lactic acid was used for enumeration of yeasts and molds, and De Man Rogosa and Sharp (7543A, Acumedia, Lansing, Michigan, United States) supplemented with nystatin (400,000 IU L^–1^) was used for enumeration of LAB. The plates were incubated aerobically at 30°C for 48 h before counting yeasts and LAB, and for 72 h before counting molds. Colony-forming units (cfu) were counted and expressed as log10.

For analyses of fermentation products, the aqueous extracts were centrifuged at 10,000 × g for 15 min at 4°C. Lactic acid (Pryce, 1969) and NH_3_-N (Chaney and Marbach, 1962) were analyzed by colorimetric methods. Volatile fatty acids, alcohols, and esters were determined by gas chromatography-mass spectrometry (GCMS QP 2010 plus, Shimadzu, Kyoto, Japan) using a capillary column (Stabilwax, Restek, Bellefonte, PA; 60 m, 0.25 mm ø, 0.25 μm crossbond carbowax polyethylene glycol). Compounds were identified based on their retention time and mass spectra and quantified with external standards (Lazzari et al., 2021).

Other silage sub-samples were dried at 60°C for 72 h. Dried samples (∼40 g) were ground in a Wiley mill through 1-mm screen and analyzed for absolute dry matter (DMoven; Association of Official Analytical Chemistry [AOAC], 1990; method 934.01), soluble carbohydrates (SC; Hall et al., 1999), crude protein (CP; Association of Official Analytical Chemistry [AOAC], 1990; method 984.13), ash (Association of Official Analytical Chemistry [AOAC], 1990; method 942.05), ether extract (EE; Association of Official Analytical Chemistry [AOAC], 1990; method 945.16), neutral detergent fiber (aNDF; assayed with a heat stable α-amylase and sodium sulfite, and expressed inclusive of residual ash; Mertens, 2002), and acid detergent fiber (ADF; Van Soest, 1963). The content of non-fiber carbohydrate was calculated as follows:

$$\text{NFC}\left(g{\mathrm{kg}}^{-1}\right)=100-\left(\text{CP}+\mathrm{aNDF}+\mathrm{EE}+\mathrm{ash}\right)$$

The DM_oven_ was corrected for loss of volatile compounds during drying, as follows (Weissbach, 2009):

DM(gkgFM-1)corr=DM(gkgFM-1)oven+n-alcohols(gkgFM-1)+2,3-butanediol(gkgFM-1)+esters(gkgFM-1)+0.95×volatilefattyacids(gkgFM-1)+0.77×1,2-propanediol(gkgFM-1)+ 0.08×lacticacid(gkgFM-1)

The n-alcohols included methanol, ethanol, and propanol; esters included ethyl lactate, ethyl acetate, and propyl acetate; and volatile fatty acids included acetic, propionic, i-butyric, butyric, i-valeric, and valeric acids.

*In vitro* DM digestibility was determined using a Daisy II incubator (ANKOM Technology, Macedon, United States). The solutions were prepared according to Tilley and Terry (1963) and the rumen fluid was obtained from two cannulated Holstein cows grazing Bermuda grass, 1 h after supplementation with 2 kg d^–1^ of concentrate based on corn, soybean meal and mineral-vitamin mix.

### Aerobic Stability Test

Silage samples (∼1 kg) were loosely placed in plastic jars insulated with polystyrene and exposed to air for 10 days in a room with controlled temperature (25 ± 2°C). Silages were sampled daily (10 g d^–1^) for measuring pH. Samples were collected without disturbing the silage mass, at approximately 10 cm deep. Temperature was recorded automatically every 15 min with a data logger inserted in the mass at approximately 15 cm deep. Simultaneously, two data loggers recorded the ambient temperature. Aerobic stability was denoted as the length of time that elapsed before silage and ambient temperatures differed by more than 2°C (O’Kiely, 1993).

### Statistical Analysis

Data were analyzed using the Mixed procedure of SAS (v. 9.4, SAS Institute Inc., Cary, NC), as a completely randomized design. Silage pH during aerobic exposure was analyzed as repeated measures, including the effect of day of exposure in the previous model. The effect of silo nested with treatment was used as error term. A first-order autoregressive covariance structure was defined because it resulted in the lowest corrected Akaike information criterion. Means were compared by Tukey-Kramer test (α = 0.05). Pearson correlations between acetic acid concentration and yeast count, ethanol concentration, gas production, DM loss, and aerobic stability were stablished using the Corr procedure of SAS.

## Results

Silage treated with LB and LH+LB had higher (*P* < 0.01) DM_oven_ and DM_corr_ compared to LH and control silages (Table 1). Silage pH at opening was relatively low in all treatments, but LH alone or associated with LB led to lowest pH (*P* < 0.01), whereas LB had an intermediate value. Overall, obligate heterofermentative LAB resulted in silages with higher contents of acetic acid, 2,3-butanediol and lower contents of ethanol, methanol, 1-propanol, and ethyl lactate compared to control (*P* < 0.01). The LB and LB+LH silages had higher concentrations of 1,2-propanediol than LH and control silages (*P* < 0.01). Ethyl acetate concentration decreased in treated silages compared to control, but LB and LB+LH silages had lowest concentrations (*P* < 0.01). Ammonia-N, lactic acid, propionic acid, butyric acid, i-butyric acid, i-valeric acid, valeric acid and mold counts were similar among treatments (*P* ≥ 0.11). The LH had lower counts of LAB than LB or untreated silage, whereas LH+LB showed intermediate counts of LAB (*P* < 0.01). Yeast counts were reduced in silages inoculated with heterolactic strains, but LH had slightly higher count of yeasts than silage inoculated with *L. buchneri* alone or in combination with *L. hilgardii* (*P* < 0.01). Acetic acid concentration was negatively correlated with yeast count (*r* = −0.76), ethanol concentration (*r* = −0.94), gas production (*r* = −0.96), and DM loss (*r* = −0.87), and positively correlated with aerobic stability (*r* = 0.60) (*P* < 0.01).

**TABLE 1:** Fermentation profile and microbial counts in sugarcane silage treated with heterolactic inoculants and stored for 70 days.

Item	Control	LH	LB	LH+LB	SEM	*P*-value
DM_oven_ (g kg^–1^ as fed)	213^c^	264^b^	284^a^	289^a^	3.80	<0.01
DM_corr_ (g kg^–1^ as fed)	253^c^	285^b^	303^a^	307^a^	3.7	<0.01
pH	3.57^a^	3.46^c^	3.48^b^	3.45^c^	0.006	<0.01
NH_3_-N (g kg^–1^ N)	60.4	62.2	65.5	62.3	2.28	0.48
Lactic acid (g kg^–1^ DM_corr_)	29.2	30.7	29.9	29.0	2.09	0.94
Ethanol (g kg^–1^ DM_corr_)	126^a^	15.6^b^	10.5^b^	7.55^b^	3.83	<0.01
Acetic acid (g kg^–1^ DM_corr_)	21.6^b^	39.2^a^	38.4^a^	39.9^a^	1.39	<0.01
2,3-Butanediol (g kg^–1^ DM_corr_)	4.15^b^	9.35^a^	9.03^a^	9.15^a^	0.439	<0.01
Ethyl lactate (mg kg^–1^ DM_corr_)	1786^a^	490^b^	347^b^	241^b^	98.1	<0.01
Ethyl acetate (mg kg^–1^ DM_corr_)	550^a^	298^b^	142^c^	152^c^	32.9	<0.01
1,2-Propanediol (mg kg^–1^ DM_corr_)	192^c^	219^c^	1642^a^	754^b^	92.8	<0.01
Propionic acid (mg kg^–1^ DM_corr_)	297	299	314	269	18.6	0.43
Methanol (mg kg^–1^ DM_corr_)	50^a^	32^b^	30^b^	30^b^	2.1	<0.01
Butyric acid (mg kg^–1^ DM_corr_)	14	13	9	10	1.7	0.16
1-Propanol (mg kg^–1^ DM_corr_)	14^a^	4^b^	3^b^	4^b^	0.6	<0.01
i-Butyric acid (mg kg^–1^ DM_corr_)	3	6	4	5	0.7	0.11
i-Valeric acid (mg kg^–1^ DM_corr_)	6	6	5	4	0.8	0.62
Valeric acid (mg kg^–1^ DM_corr_)	6	6	5	4	0.8	0.31
Lactic acid bacteria (log cfu g^–1^)	4.87^a^	2.65^b^	4.84^a^	3.50^ab^	0.286	<0.01
Molds (log cfu g^–1^)	<2	<2	<2	<2	–	–
Yeasts (log cfu g^–1^)	4.42^a^	2.06^b^	<2^c^	<2^c^	0.459	<0.01

**Control, without additive; LH, Lactobacillus hilgardii CNCM I-4785 at 3 × 10^5^ cfu g^–1^; LB, L. buchneri NCIMB 40788 at 3 × 10^5^ cfu g*^–^*^1^; LH+LB, Lactobacillus hilgardii CNCM I-4785 at 1.5 × 10^5^ cfu g*^–^*^1^ + L. buchneri NCIMB 40788 at 1.5 × 10^5^ cfu g*^–^*^1^; SEM, standard error of the mean. ^*a,b,c*^Means within a row with different superscripts differ (Tukey test, α = 0.05).**

**FIGURE 1:**
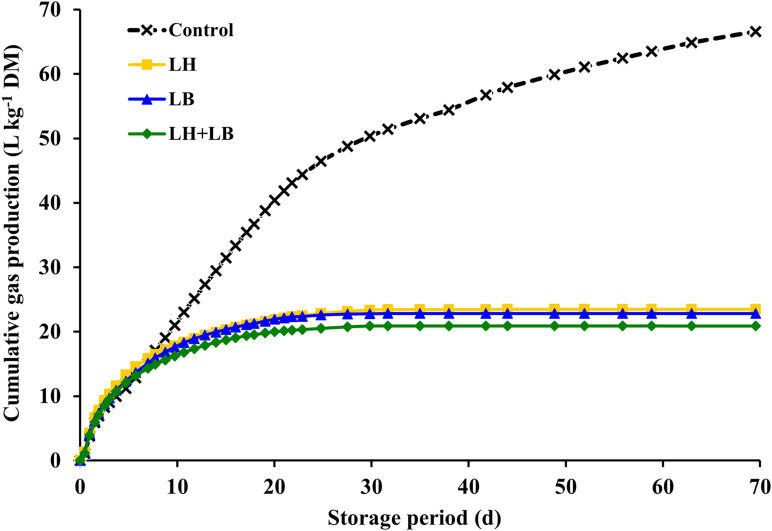
Gas production during fermentation of sugarcane silage treated with heterofermentative inoculants. Control, without additive; LH, *Lactobacillus hilgardii* CNCM I-4785 at 3 × 10^5^ cfu g^–1^; LB, *L. buchneri* NCIMB 40788 at 3 × 10^5^ cfu g^–1^; LH+LB: *Lactobacillus hilgardii* CNCM I-4785 at 1.5 × 10^5^ cfu g^–1^ + *L. buchneri* NCIMB 40788 at 1.5 × 10^5^ cfu g^–1^. Parameters of gas production kinetics are present in Table 2.

**FIGURE 2:**
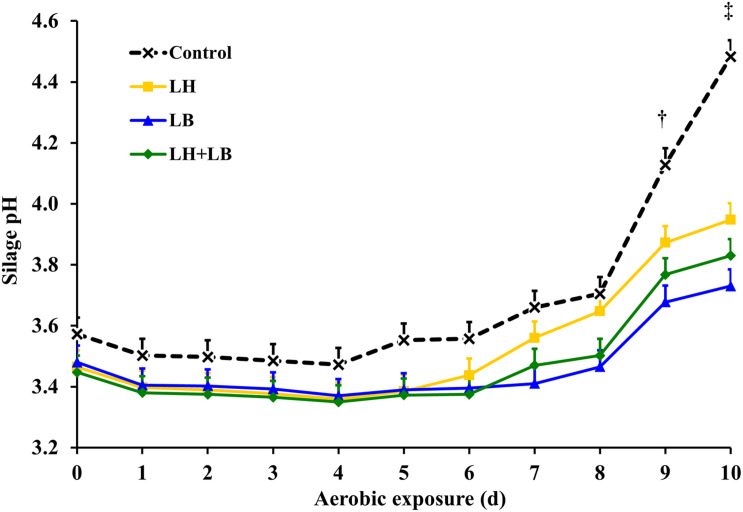
Silage pH during aerobic exposure, after 70 days of storage. Control, without additive; LH, *Lactobacillus hilgardii* CNCM I-4785 at 3 × 10^5^ cfu g^–1^; LB, *L. buchneri* NCIMB 40788 at 3 × 10^5^ cfu g^–1^; LH+LB, *Lactobacillus hilgardii* CNCM I-4785 at 1.5 × 10^5^ cfu g^–1^ + *L. buchneri* NCIMB 40788 at 1.5 × 10^5^ cfu g^–1^. *P* < 0.01 for treatment effect, *P* < 001 for day effect, and *P* < 0.01 for interaction treatment × day. Pooled standard error of the mean = 0.061. ^†^*P* < 0.05 for Control vs. LB and Control vs. LH+LB on day 9. ^‡^*P* < 0.05 for Control vs. LB, Control vs. LH and Control vs. LH+LB on day 10.

Compared with untreated silage, inoculation with obligate heterofermentative LAB consistently reduced (*P* < 0.01) gas and DM losses during fermentation (Table 2 and Figure 1). Compared to untreated silage, aerobic stability based on temperature rise was increased by the inoculation with *L. buchneri* (*P* = 0.02). Silage pH pattern during aerobic exposure indicated that LB and LH+LB delayed pH rise (*P* < 0.01) (Figure 2).

**TABLE 2:** Fermentation losses and aerobic stability of sugarcane silage treated with heterolactic inoculants and stored for 70 days.

Item	Control	LH	LB	LH+LB	SEM	*P*-value
DM_oven_ loss (g kg^–1^ DM)	335^a^	153^b^	89.2^c^	69.5^c^	11.94	<0.01
DM_corr_ loss (g kg^–1^ DM)	176^a^	56.0^b^	47.4^b^	46.2^b^	2.66	<0.01
Gas loss (g kg^–1^ DM)	129^a^	46.0^b^	43.9^b^	41.3^b^	2.20	<0.01
Gas emission^d^ (g kg^–1^ DM)	130^a^	45.9^b^	44.6^b^	40.8^b^	1.73	<0.01
Gas pool (mL kg^–1^ DM)	73.8^a^	23.1^b^	22.7^b^	20.6^b^	1.35	<0.01
Fractional rate of gas production (d^–1^)	0.040^b^	0.169^a^	0.167^a^	0.177^a^	0.0064	<0.01
Aerobic stability^e^ (h)	144^b^	143^b^	163^a^	158^ab^	4.51	0.02

**Control, without additive; LH, Lactobacillus hilgardii CNCM I-4785 at 3 × 10^5^ cfu/g; LB, L. buchneri NCIMB 40788 at 3 × 10^5^ cfu g*^–^*^1^; LH+LB, Lactobacillus hilgardii CNCM I-4785 at 1.5 × 10^5^ cfu g*^–^*^1^ + L. buchneri NCIMB 40788 at 1.5 × 10^5^ cfu g*^–^*^1^; SEM, standard error of the mean. ^*a,b,c*^Means within a row with different superscripts differ (Tukey test, α = 0.05). ^*d*^Estimated from gas volume. ^*e*^Based on temperature rise.**

Silages treated with obligate heterotactic bacteria presented lower contents of EE (*P* = 0.02), CP, aNDF and ADF (*P* < 0.01), and higher contents of NFC and SC (*P* < 0.01), as well as greater *in vitro* DM digestibility (*P* < 0.01) (Table 3).

**TABLE 3:** Chemical composition and *in vitro* digestibility of sugarcane silage treated with heterolactic inoculants and stored for 70 days.

Item	Control	LH	LB	LH+LB	SEM	*P*-value
Crude protein (g kg^–1^ DM_corr_)	25.8^a^	22.3^b^	20.9^b^	20.7^b^	0.69	<0.01
aNDF (g kg^–1^ DM_corr_)	661^a^	521^b^	497^b^	499^b^	10.8	<0.01
ADF (g kg^–1^ DM_corr_)	403^a^	306^b^	296^b^	297^b^	7.6	<0.01
Ash (g kg^–1^ DM_corr_)	35.6^a^	29.3^b^	27.5^b^	27.3^b^	0.76	<0.01
Ether extract (g kg^–1^ DM_corr_)	12.4^a^	9.48^b^	10.1^b^	10.4^b^	0.59	0.02
Non-fiber carbohydrates (g kg^–1^ DM_corr_)	266^b^	418^a^	445^a^	443^a^	11.1	<0.01
Soluble carbohydrates (g kg^–1^ DM_corr_)	213^b^	355^a^	378^a^	374^a^	9.28	<0.01
*In vitro* DM digestibility (g kg^–1^ DM_corr_)	535^b^	619^a^	629^a^	629^a^	7.48	<0.01

**Control, without additive; LH, Lactobacillus hilgardii CNCM I-4785 at 3 × 10^5^ cfu/g; LB, L. buchneri NCIMB 40788 at 3 × 10^5^ cfu g*^–^*^1^; LH+LB, Lactobacillus hilgardii CNCM I-4785 at 1.5 × 10^5^ cfu g*^–^*^1^ + L. buchneri NCIMB 40788 at 1.5 × 10^5^ cfu g*^–^*^1^; SEM, standard error of the mean. ^*a,b*^Means within a row with different superscripts differ (Tukey test, α = 0.05).**

## Discussion

Mature sugarcane is recognized by its high SC concentration at harvest (>350–400 g kg^–1^ of sucrose on DM basis; Cabezas-Garcia et al., 2011); but ensiling sugarcane can decrease its nutritive value due to the consumption of SC, mainly by yeast metabolism (Pedroso et al., 2005; Ávila et al., 2010). Anaerobically, yeasts can convert a large proportion of SC to ethanol and CO_2_ which results in high DM loss, while ethanol does not contribute to silage conservation (McDonald et al., 1991). Although ethanol has a higher content of gross energy than carbohydrates (29.7 vs. 17.6 kJ g^–1^), replacing carbohydrates with ethanol has led to similar or even lower feed efficiency, without effecting DM intake (Randby et al., 1999; Daniel et al., 2013a). Ethanol is partially volatilized from silage (Daniel et al., 2013c; Hafner et al., 2013) and partially converted to acetate and methane in the rumen (Yoshii et al., 2005), decreasing the recovery of net energy (Daniel and Nussio, 2011). Hence, alcoholic fermentation is undesirable and the use of silage additives capable of inhibiting fungi metabolism is required to obtain sugarcane silages with suitable nutritive value (Pedroso et al., 2006). In the current study we showed that applying obligate heterofermentative LAB, alone or in combination, consistently improved sugarcane silage preservation, by increasing acetic acid, inhibiting yeasts, decreasing ethanol and gas loss, sparing SC and improving *in vitro* digestibility.

The strains tested in our study are recognized by their capacity to increase acetic acid concentration in different silage types (Kleinschmit and Kung, 2006; Ávila et al., 2014; Drouin et al., 2019; Gomes et al., 2019; da Silva et al., 2021; Ferrero et al., 2021), by producing lactic and acetic acids from SC (Kandler, 1983; McDonald et al., 1991; Pahlow et al., 2003) or converting lactic acid to acetic acid and 1,2-propanediol (Oude Elferink et al., 2001). Usually, a lower lactic acid concentration is likely expected in silages treated with obligatory heterofermentative LAB (Kleinschmit and Kung, 2006). In our study, acetic acid concentration was increased by 81% (on average) in silages treated with obligate heterofermentative LAB compared to control, whereas the concentration of lactic acid was not modified by the inoculants. It may suggest that lactic acid may have been produced in a lesser extent at the onset of fermentation in control silage whereas in treated silages lactic acid was partially converted to acetic acid and 1,2-propanediol. In fact, control silage had a higher pH value compared with inoculated silages and, although acetic acid has a greater pka than lactic acid (4.8 vs. 3.8), the greater concentration of acetic acid in treated silages might have contributed to pH drop, particularly due to the low buffering capacity in sugarcane crop (Custódio et al., 2016).

Contrary to previous studies that evaluated *L. hilgardii* in sugarcane silage (e.g., Carvalho et al., 2015), corn silage (e.g., Drouin et al., 2019; Ferrero et al., 2021) or high moisture corn (e.g., da Silva et al., 2021), in this work only silage inoculated with *L. buchneri* had significant concentration of 1,2-propanediol. It strongly suggests that *L. hilgardii* produced most acetic acid by fermenting sugars (SC → lactic acid + acetic acid) whereas *L. buchneri* converted lactic acid to acetic acid and 1,2-propanediol. The concentration of 1,2-propanediol in LH silage was similar to the control and the combination LH+LB (half dose of each strain) resulted in a concentration of 1,2-propanediol that approach half of that concentration found in silage treated with a full dose of LB. Meanwhile, the content of acetic acid in LB exceeding that in control is close to the content of 1,2-propanediol in LB exceeding that in control, which support the ratio of approximately 0.5 mol of acetic acid to 0.5 mol of 1,2-propanediol converted from 1 mol of lactic acid reported by Oude Elferink et al. (2001). Hence, it seems that *L. buchneri* is more dependent on lactic acid preformed (during fermentation) as substrate whereas *L. hilgardii* prefers sugars to produce acetic acid, at least under the current conditions of strict anaerobiosis (gas-tight silos) with surplus of SC.

Acetic acid is a powerful antifungal compound capable of inhibit yeast metabolism during fermentation (anaerobiosis) and after feedout (aerobiosis) (Moon, 1983; Danner et al., 2003). In our study, acetic acid concentration was negatively correlated with yeast count, ethanol concentration, gas production and DM loss, and positively correlated with aerobic stability. In cereal crops (e.g., corn silage and small grain silages) inoculation with obligate heterofermentative LAB usually resulted in greater DM loss during fermentation by <1–6%-unit (Weinberg and Muck, 1996; Kleinschmit and Kung, 2006; Gomes et al., 2019). However, in sugarcane silages the inoculation with obligate heterofermentative LAB decreases gas loss during fermentation, because of the total amount of CO_2_ released as a byproduct of acetic acid formation is by far lower than the amount of CO_2_ released during ethanolic fermentation. In this study, obligate heterofermentative LAB not only decreased gas loss or gas emission by 66% but also curtailed the period of gas production. In control silage, 90% of gas pool was achieved at 58 days of fermentation whereas in inoculated silages that was achieved in 2 weeks (13 days). It means that after 2 weeks, the obligate heterofermentative LAB were very effective to inhibit yeast anaerobic metabolism in sugarcane silage.

As expected, the formation of low molecular weight esters was diminished in treated silages, due to the fact that concentration of ethyl esters is positively correlated with ethanol concentration (Hafner et al., 2014; Weiss, 2017). Under acidic conditions, ethanol condenses with acetic acid generating ethyl acetate, whereas ethyl lactate is formed by the esterification of ethanol on lactic acid (Hangx et al., 2001). In this way, the concentration of ethyl acetate was numerically higher in LH than in LB or LH+LB, certainly because the numerically higher concentration of ethanol in LH silage. Although there is a claim that low molecular weight esters could impair the voluntary feed intake (Weiss et al., 2016; Gerlach et al., 2019) reported no negative effects of ethyl esters (ethyl acetate and ethyl lactate) on DM intake and feed preference by goats. Meanwhile, esters are highly volatile and associated with poorer air condition in farms feeding silage-based total mixed rations (Hafner et al., 2013).

Although found at very low concentrations, methanol and 1-propanol were decreased in silages treated with obligate heterofermentative LAB. In silages, methanol is likely a product of pectin demethylation (Steidlová and Kalac, 2002; Parra et al., 2019), due to the activity of pectinolytic enzymes, which are widely distributed in nature and are produced by yeast, bacteria, fungi and plants (Sieiro et al., 2012). Dato et al. (2005) reported that contaminating yeasts produced methanol during fermentation of sugarcane juice to produce *cachaça* (an alcoholic beverage), which has a fermentation pattern relatively similar to that in sugarcane silage. Therefore, a higher yeast metabolism might have increased pectin demethylation and methanol formation in control silage.

1-Propanol in silage can be produced by yeasts (McDonald et al., 1991; Giudici et al., 1993), clostridia (Janssen, 2004) and LAB such as *Lentilactobacillus diolivorans* (basonym *Lactobacillus diolivorans*) capable of converting 1,2-propanediol to propionic acid and 1-propanol (Krooneman et al., 2002). It is unlikely that *L. diolivorans* had developed substantially in this study, as our silages had low concentrations of propionic acid and 1-propanol, even in that cases where 1,2-propanediol was abundant. Also, low concentration of butyric acid (<14 mg kg^–1^ DM_corr_) indicated that *Clostridium* sp. was suppressed during sugarcane silage fermentation. Therefore, 1-propanol might have been derived of yeast activity, as greater concentration of 1-propanol was observed in untreated silage.

Interestingly, all inoculated silages presented more 2,3-butanediol than control. The 2,3-butanediol can be produced by different microorganisms, such as LAB and bacillus (McDonald et al., 1991), clostridia (Siemerink et al., 2011; Fernandes et al., 2020), and enterobacteria (Rooke and Hatfield, 2003; Nishino and Shinde, 2007). Recently, Gomes et al. (2019) reported a consistently higher concentration of 2,3-butanediol in oat silages inoculated with *L. buchneri*. They attributed that greater formation of 2,3-butanediol to enterobacteria (a group of bacteria that develop under non-acidic conditions), because of higher silage pH and silage heterogeneity (Pahlow et al., 2003), due to the possible formation of ecological niches with higher pH in silages treated with *L. buchneri*. In the current study, the origin of 2,3-butanediol is less clear, because treated silages had lower pH than control. Additionally, a low conversion of lactic acid to acetic acid and 1,2-propanediol in the LH treatment (discussed above) weakens the hypothesis of ecological niches that would promote enterobacteria development (Pahlow et al., 2003). Further studies are warranted to explain why 2,3-butanediol may increase in silage inoculated with obligate heterofermentative LAB.

In sugarcane silages, aerobic deterioration is often initiated by lactate-utilizing yeasts, which result in silage heating and pH rise, enabling the proliferation of fungi and aerobic bacteria less tolerant to acidity (Ávila et al., 2012). Acetic acid bacteria can also be involved in aerobic deterioration of sugarcane silage (Daniel, unpublished). In the current trial, a slight decay of silage pH after 24 h of aerobic exposure suggests that ethanol might have been partially oxidized to acetic acid (Spoelstra et al., 1988).

Meanwhile, the rate of aerobic deterioration is driven by a combination of factors such as the initial counts of spoiling microorganisms, substrate availability (mainly SC and lactic acid) and presence of inhibitory substances, such as acetic acid (Danner et al., 2003). In the current study, *L. buchneri* and *L. hilgardii* strains increased acetic acid concentration and spared SC in a similar degree, but *L. buchneri* was the most effective strain to improve aerobic stability, based on a collective interpretation of temperature and pH rise during the aerobic stability test. On the other hand, the time elapsed for temperature rise in LH silage was similar to that in control, probably due to the greater yeast population present in LH treatment compared with *L. buchneri*. Hence, the combination of LH+LB (half dose of each strain) resulted in intermediate values of aerobic stability based on temperature rise. Collectively, those results support the idea that silage aerobic stability is not only a function of acetic acid concentration or substrate availability for aerobic microorganisms. Also, the fungal communities may have shifted from lactate to non-lactate utilizing yeasts, as recently reported by da Silva et al. (2020) in corn silage treated with chemical additives.

Our results of aerobic stability for the LH treatment contrasted with those reported by Ávila et al. (2014) and Carvalho et al. (2015) who described longer stability in sugarcane silages treated with *L. hilgardii* compared to untreated silage. However, Rabelo et al. (2019) demonstrated by meta-analysis that the effect of obligate heterofermentative LAB on the aerobic stability of sugarcane silages is not consistent. In our study, sugarcane silages (including the control) showed an unusual prolonged aerobic stability compared with previous reports. The aerobic stability of sugarcane silages in studies compiled by Rabelo et al. (2019) ranged from 30 to 100 h. The longer aerobic stability reached in our study might be due to the strictly anaerobic conditions imposed by the gas-tight silos. In fact, yeast counts were lower than 5 log cfu g^–1^ in all treatments, which is considered as threshold count for promptly aerobic deterioration (Borreani et al., 2018).

All nutrient changes observed among treatments were consequence of the consumption or sparing of SC. In treated silages, the microbial inoculants spared SC, whereas in control silage high yeast activity consumed 42% more SC in comparison with inoculated silages, increasing the concentrations of fiber (i.e., aNDF and ADF) and non-carbohydrate nutrients (i.e., CP, ash and EE) by a dilution effect. As the true digestibility of the organic matter soluble in neutral detergent solution (i.e., NFC, CP and EE) is almost complete (Van Soest, 1967; Daniel et al., 2017), DM digestibility is primarily a function of the concentration and digestibility of aNDF. The sugarcane crop used in this study was harvested from the same field and obviously had similar aNDF digestibility among treatments. Therefore, silages with lower aNDF were highly expected to have the greater observed *in vitro* DM digestibility. Other studies evaluating obligate heterofermentative LAB in sugarcane silage have been less consist, with higher (Andrade et al., 2016), equal (Pedroso et al., 2007, 2008) or even lower DM digestibility in treated silage compared with that in control (Rabelo et al., 2019). Differences of inoculant dose among studies (often lower than used in the current study) might partially explain those divergent findings.

## Conclusion

All heterolactic inoculants applied at 3 × 10^5^ cfu g^–1^ were effective to inhibit yeast metabolism and mitigate gas loss during fermentation resulting in sugarcane silages with greater nutritional value. Nevertheless, *L. buchneri* strain 40788 was the most effective strain to extend the aerobic stability of sugarcane silage in addition to the improved nutrient recovery during fermentation. Our findings do not support the requirement of crop specific strains of obligate heterofermentative LAB to improve the conservation of sugarcane silage.

## Data Availability Statement

The raw data supporting the conclusions of this article will be made available by the authors, without undue reservation.

## Author Contributions

All authors contributed equally to this manuscript.

## Conflict of Interest

The authors declare that the research was conducted in the absence of any commercial or financial relationships that could be construed as a potential conflict of interest.
